# Diastereomers of the Brominated Flame Retardant 1,2-Dibromo-4-(1,2 dibromoethyl)cyclohexane Induce Androgen Receptor Activation in the HepG2 Hepatocellular Carcinoma Cell Line and the LNCaP Prostate Cancer Cell Line

**DOI:** 10.1289/ehp.0901065

**Published:** 2009-08-03

**Authors:** Hazem Khalaf, Anders Larsson, Håkan Berg, Robert McCrindle, Gilles Arsenault, Per-Erik Olsson

**Affiliations:** 1 Örebro Life Science Center, Academy of Science and Technology, Örebro University, Örebro, Sweden; 2 Wellington Laboratories Inc., Research Division, Guelph, Ontario, Canada

**Keywords:** androgen, brominated flame retardant, endocrine disruptor

## Abstract

**Background:**

Reported incidences of prostate cancer and masculinization of animals indicate a release of compounds with androgenic properties into the environment. Large numbers of environmental pollutants have been screened to identify such compounds; however, not until recently was 1,2-dibromo-4-(1,2-dibromoethyl)cyclohexane (TBECH) identified as the first potent activator of the human androgen receptor (hAR). TBECH has been found in beluga whales and bird eggs and has also been found to be maternally transferred in zebrafish.

**Objectives:**

In the present study we investigated interaction energies between TBECH diastereomers (α, β, γ, and δ) and the hAR, and their ability to activate the receptor and induce prostate-specific antigen (PSA) expression *in vitro*.

**Methods:**

We performed computational modeling to determine interaction energies between the ligand and the AR ligand-binding site, and measured *in vitro* competitive binding assays for AR by polarization fluorometry analysis. We used enzyme-linked immunosorbent assays to determine PSA activity in LNCaP and HepG2 cells.

**Results:**

We found the γ and δ diastereomers to be more potent activators of hAR than the α and β diastereomers, which was confirmed in receptor binding studies. All TBECH diastereomers induced PSA expression in LNCaP cells even though the AR present in these cells is mutated (T877A). Modeling studies of LNCaP AR revealed that TBECH diastereomers bound to the receptor with a closer distance to the key amino acids in the ligand-binding domain, indicating stronger binding to the mutated receptor.

**Conclusions:**

The present study demonstrates the ability of TBECH to activate the hAR, indicating that it is a potential endocrine disruptor.

During the last several years, the incidences of prostate and testicular cancer have increased significantly. By 50 years of age, about 50% of all men will suffer from prostatic hyperplasia (Berry et al. 1984). It has been demonstrated that exposure to androgens, such as dihydrotestosterone (DHT), increases the risk for the development of prostate cancer (Carson and Rittmaster 2003). There have also been reports of masculinization of animals and wildlife populations (Ellis et al. 2003). The increase in prostate cancer and the recorded masculinization of animals indicate that there are compounds in the environment with androgenic properties. This has led to research focused on the identification of substances with potential AR agonistic properties by screening large numbers of environmental compounds (Fang et al. 2003; Kojima et al. 2004; Sohoni and Sumpter 1998). These studies led to the identification of several estrogen receptor (ER) agonists as being androgen antagonists. However, although an earlier study demonstrated that 2-*tert*-butylanthraquinone and benzoanthrone may act as weak agonists to the human androgen receptor (hAR) at high concentrations (Araki et al. 2005), only recently did our group identify the brominated flame retardant (BFR) 1,2-dibromo-4-(1,2 dibromoethyl)cyclohexane (TBECH) as the first environmental chemical to bind to and activate the hAR with high potency (Larsson et al. 2006). AR is activated through binding of a ligand, such as testosterone or DHT, to its ligand-binding domain (LBD), followed by dissociation of inhibitory heat-shock proteins. After activation, the AR–ligand complex migrates into the nucleus and binds to its response element, which together with coactivators initiates transcription and cellular responses (Brinkmann et al. 1999; Veldscholte et al. 1992).

Because of the four chiral carbons present in its structure, TBECH can exist in four diastereoisomeric forms (α, β, γ, and δ). The α and β forms are found in the commercial flame retardant Saytex BCL 462 (Albemarle Corp., Baton Rouge, LA, USA), whereas the γ and δ forms are converted from α and β at temperatures > 120°C (Arsenault et al. 2008). In 2002, TBECH was reportedly produced at volumes between 4 and 225 metric tons [U.S. Environmental Protection Agency (EPA) 2002]. TBECH was reported to be mutagenic in a study that tested 27 different chemicals using a L5178Y tk^+^/tk^−^ mouse lymphoma-cell forward-mutation assay (McGregor et al. 1991). In 1995, TBECH was found to be present in industrial waste water near Haifa, Israel (Santillo et al. 1997), and more recent studies have reported the presence of TBECH in beluga whales in the Canadian Arctic (Tomy et al. 2008). In a recent study using zebrafish, Nyholm et al. (2008) showed that TBECH can be maternally transferred; they speculated that higher levels of TBECH would be found in the offspring of species that invest more lipids in their eggs, which is in line with the recent discovery of TBECH in eggs from herring gulls and double-crested cormorants (Gauthier et al. 2009).

In the present study, we analyzed the interaction energies between the different TBECH diastereomers and the hAR *in silico* and determined their potential to bind and activate the receptor and its downstream target, prostate-specific antigen (PSA) *in vitro*.

## Materials and Methods

### Chemicals

We synthesized TBECH diastereomers as previously described (Arsenault et al. 2008). DHT and testosterone were purchased from Sigma Aldrich (St. Louis, MO, USA). TBECH-αβ and TBECH-β were dissolved with dichloromethane that was allowed to evaporate after addition of dimethyl sulfoxide (DMSO). All other ligands were dissolved in DMSO. Exposure of cells was performed in cell culture media with a maximum of 0.1% DMSO present.

### Computational modeling

We determined the interaction energies between the ligand and the AR ligand-binding site using the Molecular Operating Environment (MOE) program (Chemical Computing Group, Köln, Germany). The crystal structure of the hAR obtained from the Protein Data Bank entry 1e3g (PDB 2009) was subjected to minimization using the Amber99 force field embedded in MOE, and the minimized structure was used as template for dockings with all ligands. Before docking, the ligand-binding site was determined using the MOE Alpha Site Finder. We performed the dockings as Monte Carlo–simulated annealing-based flexible docking of the ligands into the receptor, using the automated docking as incorporated in MOE. Each evaluated system was set to a maximum of 500 confirmed dockings, where the docked structures were sorted based on the lowest *S*-value (the objective function, based on evaluating the affinity Δ*G* scoring function, which is a combination of strain energy and mutual similarity score). Before calculation of interaction energies, the docked structures were subjected to relaxation, molecular dynamics simulations, and additional relaxation as previously described (Larsson et al. 2006). The AR from LNCaP cells (a prostate cancer cell line) harbors a mutation (T877A) in the ligand-binding pocket (LBP), so the LNCaP AR was modeled using the crystal structure of the hAR as a template, as previously described (Larsson et al. 2006). The model was generated as a Cartesian average of 10 models and minimized using the Amber99 force field. When this was done, the LNCaP AR model was used for docking simulations in the same manner as the hAR.

### Competition assay

We performed competitive binding assays for AR using the PolarScreen AR competition assay (PanVera, Madison, WI, USA) according to the manufacturer’s instructions, using polarization fluorometry analysis, with excitation at 485 nm and emission at 535 nm, on a GeniosPro instrument (Tecan Trading AG, Männedorf, Switzerland). The AR-LBD used in the PolarScreen AR competition assay is derived from rat but shows 100% sequence homology to the hAR: The amino acid sequences of the LBDs of rat and human AR [GeneBank accession numbers J05454 and M20132 (National Center for Biotechnology Information 2009), respectively] are identical. The final concentration of AR LBD was 50 nM. Binding affinity was determined using competition against the synthetic androgen Fluormone AL Green ligand (2 nM; Invitrogen). DHT was used as a positive control. We produced competition curves for DHT and TBECH diastereomers using concentrations ranging from 1 nM to 10 μM. All analyses were performed in triplicate.

### Cell culture, transfection, and stimulation

HepG2 hepatocellular carcinoma cells were cultured in Eagle minimal essential medium (E-MEM; Invitrogen) supplemented with 10% fetal calf serum (FCS; HyClone; Nordic Biolabs, Taby, Sweden), 1 mM sodium pyruvate (Invitrogen), 0.1 mM non-essential amino acids (Biochrom AG, Berlin, Germany), and 1% antibiotic antimycotic mixture (Invitrogen) in an incubator at a stable environment of 95% humidity, 5% CO_2_, and 37°C.

Before transfection, the cells were seeded onto 24-well plates in an antibiotic-free and phenol-free medium complemented with charcoal-stripped FCS. The charcoal-stripped serum was prepared by mixing serum with activated charcoal and Dextran T-70 (Sigma-Aldrich Sweden AB, Stockholm, Sweden). After 12 hr incubation at 4°C, the mixture was centrifuged to remove the charcoal/dextran, and the supernatant was filter-sterilized. At 90–95% confluence, the cells were transfected with 270 ng *slp*-ARE-Luc (sex-limiting protein–androgen response element–luciferase) reporter, 270 ng hAR expression vector (pCMVhAR), and 60 ng *Renilla* luciferase (pRL; Promega, Madison, WI, USA) using Lipofectamine 2000 (Invitrogen) according to the manufacturer’s recommendations. The *slp*-ARE-Luc vector contains four copies of an ARE that has been shown to be specific for AR activation while minimizing the influence of glucocorticoids (Verrijdt et al. 2002). At 24 hr posttransfection, the medium was aspirated and replaced with medium containing different concentrations DHT, testosterone, or different diastereomers of TBECH. After exposure (8 hr for testosterone and DHT, 40 hr for TBECH), the cells were lysed *in situ* using passive lysis buffer (Promega), and luciferase levels were measured using the Dual Luciferase Assay Kit (Promega) in a TD 20/20 luminometer (Turner Designs, Sunnyvale, CA, USA). The luciferase values were normalized to the corresponding *Renilla* values. All analyses were performed in triplicate.

### Enzyme-linked immunosorbent assay (ELISA)

For the ELISA, human LNCaP cells were cultured in culture flasks in E-MEM (Invitrogen) supplemented with 10% FCS, 1 mM sodium pyruvate, 0.1 mM nonessential amino acids, and 1% antibiotic antimycotic mixture in an incubator at a stable environment of 95% humidity, 5% CO_2_, and 37°C. Before challenge, the cells were seeded onto 24-well plates in cell culture media containing charcoal-stripped serum.

LNCaP cells were challenged with 100 nM DHT or different combinations of the TBECH diastereomers at 1 μM. The cells were challenged for 5 days; the supernatants were collected by gentle pipetting and stored at −80° C until use. PSA quantification was performed by coating each well, in 96-well plates, with 100 μL of a 1:1 mix of sample and coating buffer (0.1 M sodium carbonate, pH 9.6) followed by incubation for 1 hr at room temperature. To obtain a standard curve, serial dilutions of human PSA (Sigma, St. Louis, MO, USA) were prepared, loaded onto the plates, and treated like the samples. The buffer was aspirated, and each well was washed 3 times with 300 μL wash buffer [phosphate-buffered saline (PBS) with 0.05% Tween-20, pH 7.4]. This was followed by the addition of 200 μL blocking buffer (PBS with 5% bovine serum albumin) and incubation for 1 hr at room temperature. The plates were then washed 3 times with 300 μL wash buffer and incubated with 100 μL primary antibody (anti-human PSA; Sigma) for 1 hr at room temperature. The plates were washed and incubated with 100 μL secondary antibody (ECL anti-rabbit, horseradish peroxidase–linked whole antibody (Amersham Biosciences, Little Chalfont, Buckinghamshire, UK) for 30 min at room temperature. Detection was performed by adding 100 μL 1-Step Turbo TMB-ELISA (Pierce, Rockford, IL, USA) for 30 min. The reaction was stopped with 50 μL 1 M H_2_SO_4_, and the absorbance was measured at 450 nm using a Multiskan MS plate reader (Thermo Labsystems, Vantaa, Finland).

### Statistical analysis

Statistical significant differences were determined using two-tailed Student’s *t*-test.

## Results

### Ligand docking

**Figure 1:**
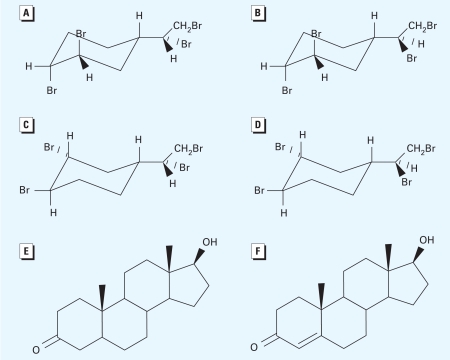
The molecular structures of the BFRs examined, along with structures of DHT and testosterone for comparison. (*A*) TBECH-α{ *rac*-(1*R*,2*R*)-1,2-dibromo-(4*S*)-4-[(1*R*)1,2-dibromoethyl]cyclohexane}. (*B*) TBECH-β {*rac*-(1*R*,2*R*)-1,2-dibromo-(4*S*)-4-[(1*S*)1,2-dibromoethyl]cyclohexane}. (*C*) TBECH- γ {*rac*-(1*R*,2*R*)-1,2-dibromo-(4*R*)-4-[(1*R*)1,2-dibromoethyl]cyclohexane}. (*D*) TBECH-δ {*rac*-(1*R*,2*R*)-1,2-dibromo-(4*R*)-4-[(1*S*)1,2-dibromoethyl]cyclohexane}. (*E*) DHT. (*F*) Testosterone.

We determined ligand docking of TBECH in hAR and LNCaP AR LBD using molecular modeling techniques. The BFR TBECH can exist as four diastereomers (α, β, γ, and δ), which differ in the manner in which the bromine atoms are oriented in their molecular structures (Figure 1). These different orientations of the bromine atoms in the molecule strongly affect their binding affinities in the AR LBD.

**Figure 2:**
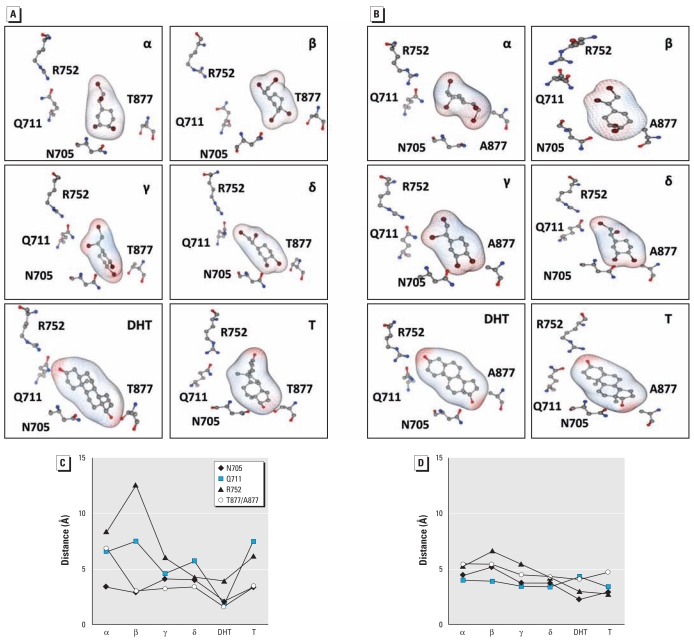
The four key amino acids (N705, Q711, R752, and T877/A877) in the active site of the hAR (*A*) and LNCaP AR (*B*) shown with the natural ligands DHT, testosterone (T), and the four TBECH diastereomers (*C* and *D*). The closest distances between the amino acids and the ligands for hAR (*C*) and for LNCaP AR (*D*). See “Materials and Methods” for details of simulations.

Docking simulations established that all four TBECH diastereomers occupied the same hAR LBP as did DHT (Figure 2A). In the LNCaP AR, the structure of the LBP differs from the hAR because of the T877A mutation. This mutation shortens the distance between ligands and Ala877 in the LBP with a concomitant reduction of ligand discrimination (Figure 2B). The differences in distance between the key amino acids [Asn705 (N705), Gln711 (Q711), Arg752 (R752), Thr877 (T877), and Ala877 (A877)] and ligands are shown in Figure 2C and D.

### Ligand–receptor interaction energies

The substantial differences in distances between the amino acids in the LBP and ligand observed for the four TBECH diastereomers manifested as significant differences in interaction energies. The natural ligands DHT and testosterone bound to the hAR with interaction energies of −53.8 and −48.9 kcal/mol, respectively (Table 1). Among the four TBECH diastereomers, TBECH-δ displayed the closest similarities to DHT (Table 2, Figure 2C). The LBP interaction energy with TBECH-δ was −40.1 kcal/mol, whereas those for TBECH-α, -β, and -γ were −34.8, −26.8, and −35.3 kcal/mol, respectively. Interaction of DHT and TBECH diastereomers with the key amino acids in the hAR LBD are shown in Figure 2A and C. In the LNCaP AR, the mutation T877A resulted in increased interaction energies, which suggest stronger interactions with the key amino acids in the mutated receptor (Tables 1 and 2, Figure 2B,D).

### Competition assays

**Figure 3:**
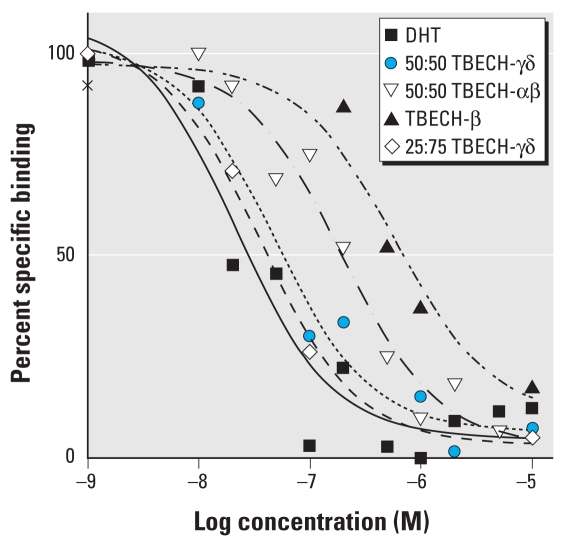
Competition curves for binding of DHT 50:50 TBECH-γδ, 50:50 TBECH-αβ, and purified TBECH-β to the rat AR. The amino acid sequences of the LBDs of rat and human AR are identical, so the results can be extrapolated to hAR. Each value represents the mean of three assays.

We tested the different diastereomers of TBECH for receptor binding affinity using the PolarScreen AR competitive assay. In contrast to TBECH-β, TBECH diastereomers α, γ, and δ cannot be isolated; this prompted analysis of combinations of these in the receptor binding assay. We determined the binding affinity using competition against dexamethasone, (Figure 3). In this assay, DHT had a half-maximal inhibitory concentration (IC_50_) of 21.5 nM with a goodness of fit (*R*^2^) of 0.91. The relative affinity of the TBECH diastereomers was 655 nM (*R*^2^ = 0.96) for TBECH-β, 191 nM (*R*^2^ = 0.97) for a 50:50 mixture of TBECH-α and -β (TBECH-αβ), 47.4 nM (*R*^2^ = 0.98) for a 50:50 mixture of TBECH-γ and -δ (50:50 TBECH-γδ), and 35.9 nM (*R*^2^ = 0.99) for a 25:75 mixture of TBECH-γδ. This indicates that TBECH-γδ binds AR with an affinity very similar to that of DHT, whereas TBECH-β has the lowest affinity of the TBECH diastereomers (Figure 3).

### AR activation assays

We determined the activation capacity of the different TBECH diastereomers using transactivation studies in HepG2 and LNCaP cells. We used HepG2 cells to allow comparison with an earlier study (Larsson et al. 2006), and we used LNCaP cells because they contain an AR mutation that is frequently seen in prostate cancer. Before exposure, the cells were transfected with the *slp*-ARE-Luc reporter vector and the hAR expression vector pCMVhAR together with an internal control (pRL). We used the *slp*-ARE-Luc vector because it contains four copies of an ARE that is highly specific for AR interactions. The optimal time and concentration of exposure was determined for DHT, testosterone, and different combination of the TBECH diastereomers β, 50:50 αβ, 50:50 γδ, and 25:75 γδ.

**Figure 4:**
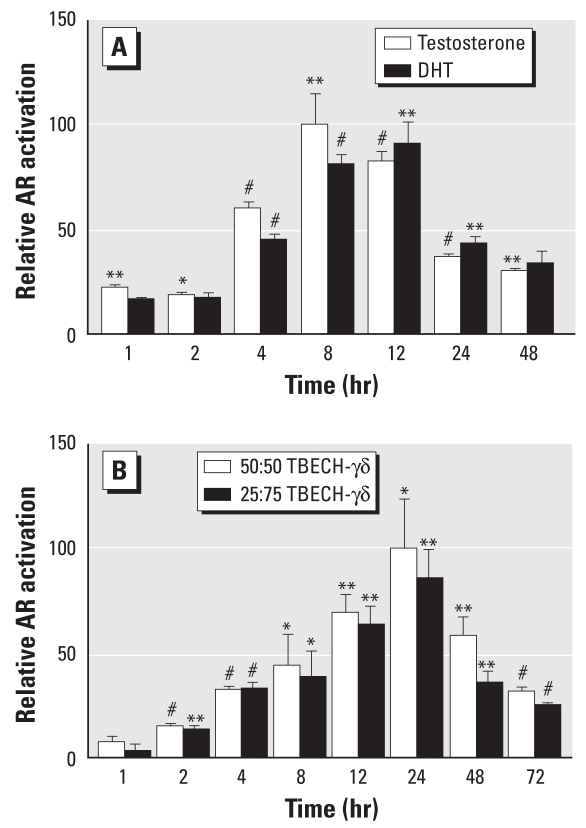
*In vitro* analysis of AR activation using HepG2 cells were transfected with both the *slp*-ARE-Luc reporter vector and AR expression vector pCMVhAR. (*A*) Time-dependent activation of AR after exposure to 100 nM T and 10 nM DHT. (*B*) Time-dependent activation of AR after exposure to 50:50 TBECH-γδ or 25:75 TBECH-γδ at a final concentration of 1 μM. All values were normalized against the controls; the control levels were arbitrarily set to 1, and maximal induction was set to 100%. *n*= 4 per exposure group. *p < 0.05, **p < 0.01, and ^#^*p* < 0.001 by Student’s *t*-test compared with controls.

Stimulation of HepG2 cells with testosterone and DHT resulted in maximal induction after 8–12 hr of exposure (Figure 4A), whereas the TBECH-γδ diastereomers (both 50:50 and 25:75) showed a slower response with maximal induction after 24 hr (Figure 4B). DHT was also a stronger inducer than was testosterone, which required 10-fold higher concentrations (100 nM) to induce hAR activation to the same level as DHT.

AR activity decreased after further stimulation at 24 hr and 48 hr, suggesting early activation and fast cellular metabolism of DHT. We also assessed the time-dependent AR activation in response to different combinations of TBECH-γδ (50:50 and 25:75) using a final concentration of 1 μM. The two TBECH-γδ combinations activated the AR equally. Activation of AR reached statistical significance after 2 hr and maximal induction after 24 hr (Figure 4B).

**Figure 5:**
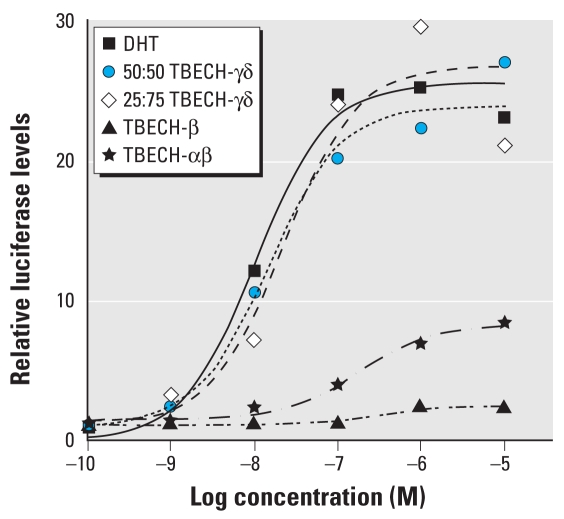
Determination of AR activation in response to TBECH diastereomers. HepG2 cells were stimulated with DHT for 8 hr or with TBECH diastereomers for 40 hr at concentrations ranging from 1 nM to 10 μM. Both combinations of TBECH-γδ were more potent AR activators than TBECH-β or TBECH-αβ. All values were normalized against the controls that were arbitrarily set to 1. *n*= 4 per exposure group.

To determine the optimal concentrations of DHT and TBECH isoforms for AR activation, we stimulated HepG2 cells with DHT in a dose-dependent manner for 8 hr and with TBECH for 40 hr. Exposure to 50:50 TBECH-γδ resulted in a half-maximal effective concentration (EC_50_) of 14.9 nM (*R*^2^ = 0.96), whereas exposure to 25:75 TBECH-γδ resulted in an EC_50_ of 22.7 nM (*R*^2^ = 0.94; Figure 5). This suggests that TBECH-γ may be a better inducer of AR than TBECH-δ. Both of these diastereomers induce AR at concentrations that are comparable to those of DHT (10.5 nM; *R*^2^ = 0.92) and indicate that these TBECH diastereomers are highly potent androgen agonists. Determination of AR activation by 50:50 TBECH-αβ demonstrated that these diastereomers are less potent, with an EC_50_ of 174 nM (*R*^2^ = 0.94), one order of magnitude higher than DHT or TBECH-γδ. The weakest inducer of AR was TBECH-β, with an EC_50_ of 294 nM (*R*^2^ = 0.84). We also observed that TBECH-γ and -δ are complete agonists to DHT, whereas TBECH-α and -β are partial agonists because they conferred only partial induction. Determination of relative induction by the different compounds showed a 23.7 ± 5.0-fold induction after exposure to DHT, a 24.4 ± 5.1-fold induction with 50:50 TBECH-γδ, a 25.5 ± 1.3-fold induction with 25:75 TBECH-γδ, an 8.4 ± 1.2-fold induction with TBECH-αβ, and a 2.4 ± 0.3-fold induction with TBECH-β.

### In vitro ELISA assays

**Figure 6:**
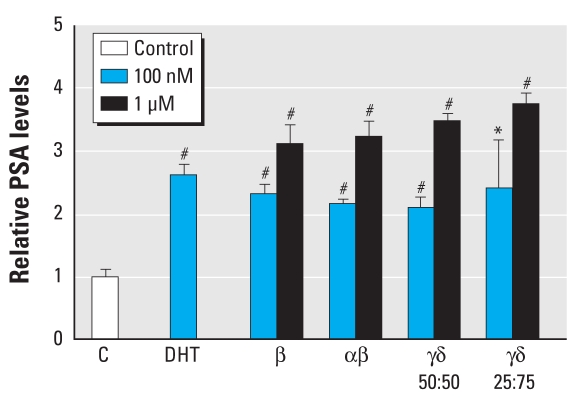
Determination of PSA expression in LNCaP cells treated with DHT or one of the four mixtures of TBECH diastereomers [TBECH-β, 50:50 TBECH-αβ, 50:50 TBECH-γδ, or 25:75 TBECH-γδ) at a final concentration of 100 nM and 1 μM for 5 days. C, control. *n*= 4 per exposure group. *p < 0.05 and ^#^*p* < 0.001 (Student’s *t*-test).

We used LNCaP cells to determine the ability of TBECH to induce endogenous gene expression of PSA, a downstream target of the AR. The results revealed a 2.61 ± 0.15-fold induction of PSA after treatment with 100 μM DHT (Figure 6). PSA expression increased in response to all four TBECH diastereomer combinations in a dose-dependent manner. Exposure to 1μM of the TBECH diastereomers resulted in a 3.11 ± 0.31-fold induction by TBECH-β, a 3.22 ± 0.26-fold induction by 50:50 TBECH-αβ, 3.47 ± 0.12-fold induction by 50:50 TBECH-γδ, and a 3.74 ± 0.17-fold induction by 25:75 TBECH-γδ. The equivalent inducibility by the different compounds is in agreement with the modeling data that show similar distances to the key amino acids in the AR LBP of the mutated LNCaP AR.

## Discussion

TBECH belongs to a group of BFRs that are found in a variety of products, for example, oriented strand boards, particle boards, and rigid foam and soft foam used in insulation and as stuffing in furnishings, respectively, or as an additive in polystyrene and polyurethane. The discovery of TBECH’s presence in both sediments and organisms (Gauthier et al. 2009; Nyholm et al. 2008; Santillo et al. 1997; Tomy et al. 2008), along with its potent activation of AR at nanomolar concentrations, suggests that these compounds constitute a serious threat to both humans and wildlife. Using the Waste Minimization Prioritization Tool, the U.S. EPA (2000) ranked TBECH as one of the 10% most hazardous compounds to ecosystems. TBECH is an additive flame retardant, blended into material during manufacturing. In some processes this may involve thermal procedures and certainly, in the event of fire, elevated temperatures are likely to cause an interconversion among the different TBECH diastereomers. Thus, although TBECH-β is the most abundant isoform, it is reasonable to believe that all four diastereomers are present and cause a threat to the environment.

A well-known characteristic of steroid receptors is that they bind their natural ligand with high specificity. Reported IC_50_ and EC_50_ values for sex steroid receptors by their natural ligands and the aryl hydrocarbon receptor (AhR) by TCDD (2,3,7,8-tetrachlorodibenzo-*p*-dioxin) are listed in Table 3. Interestingly, when investigating substances with reported high endocrine-disrupting effects such as nonylphenol or bisphenol A (both are estrogenic) and vinclozolin (an androgen antagonist), none of the substances, with the exception of TCDD, binds to or activates the steroid receptors by > 1% of the endogenous ligand (Table 4). In our study, we found DHT to have an IC_50_ of 21.5 nM, which correlates well with IC_50_ values reported in other studies (Table 3). When we examined the TBECH diastereomer binding activities, we found that 50:50 TBECH-γδ binds to the AR with 22% of DHT’s binding affinity, whereas for 50:50 TBECH-αβ we found a relative binding affinity (RBA) of 6%. This correlated well with the binding and activation studies, in which 50:50 TBECH-γδ displayed a higher activation/binding than TBECH-αβ (Figures 5 and 6). Furthermore, comparison of activation potential demonstrated that both TBECH-γδ mixtures were was as potent as DHT at activating the hAR (Figure 5). The only other environmental contaminant inferred to be able to maximally activate a ligated receptor is TCDD (Table 4). This indicates that the TBECH diastereomers are extremely potent AR agonists compared with other known pollutants with proven endocrine-disrupting effects.

Recently, TBECH was shown to be present in beluga whales and herring gull eggs at nanomolar concentrations (Gauthier et al. 2009; Tomy et al. 2008). Low-level exposure to endocrine-disrupting compounds can induce functional, developmental, behavioral, and transgenerational disturbances, as shown after low-level exposure to the fungicide vinclozolin, which acts as an androgen antagonist in rats and mice (Anway et al. 2006, 2008; Elzeinova et al. 2008; Skinner et al. 2008). Therefore, although low-level exposure to the most common TBECH, TBECH-β, may not induce high AR activation, these compounds may nonetheless induce transgenerational effects at the observed levels.

The natural ligands testosterone and DHT showed maximal hAR activation already after 8 hr (Figure 4). A recent study showed that HepG2 cells rapidly metabolized testosterone and DHT, resulting in 82% and 46% reduction, respectively, 21 hr after addition of the hormone (Simon and Mueller 2006). In contrast, determination of time-dependent hAR activation by TBECH indicates slower receptor activation and prolonged induction times, suggesting that these compounds are more stable in this cell line. The stability of TBECH is further supported by the discovery of its presence in the environment (Gauthier et al. 2009; Tomy et al. 2008) as well as its ability to be maternally transferred in zebrafish (Nyholm et al. 2008).

Although LNCaP is an androgen-dependent cell line with a mutated AR (T877A), it retains the androgen binding and ligand specificity in the LBP of the AR (Gaddipati et al. 1994; Wang et al. 1997). This mutation is frequently detected and has been reported to be present in 30% of hormone-refractory prostate cancer patients (Taplin et al. 1999). PSA is a well-defined androgen-regulated glycoprotein present in LNCaP cells and is widely used as a marker for prostate cancer diagnosis (Wang et al. 1997). In the present study, we found that both DHT and the four TBECH diastereomers induce comparable PSA expression in LNCaP cells when exposed to 100 nM of the compounds. TBECH-β was located farthest away from R752 in the hAR. This could explain its poor ability to activate the hAR. However, the LNCaP AR mutation (T877A) enables the different diastereomers to bind with a closer distance to the key amino acids in the LBP, which could explain TBECH-β’s ability to induce PSA expression in LNCaP cells. The higher affinity of TBECH-β to LNCaP AR and the frequency of the T877A mutation suggest that these compounds are active in a large proportion of prostate tumors and that they may contribute to the etiology of prostate cancer.

The present study provides important data on the ability of TBECH to bind and activate the hAR with high affinity. Combining the results from the molecular modeling, the competition assay, and the activation assay, the TBECH-δ diastereomer appears to be the most potent, followed by TBECH-γ and TBECH-α, with TBECH-β being the least potent activator of AR. Furthermore, as the modeling experiments are in agreement with results obtained from the *in vitro* studies, this demonstrates that modeling is a powerful tool when identifying potential endocrine disruptors. However, it remains unknown how TBECH interacts with AR in other species, such as three-spined stickleback and zebrafish, that both have 11-ketotestoterone as their most potent activator of the AR (Hossain et al. 2008; Olsson et al. 2005). Therefore, future studies are needed to determine the interaction of TBECH with AR from other species in order to determine its effects on species present in the environment.

## Figures and Tables

**Table 1:** Interaction energies and distances between the ligand and the key amino acids of the hAR determined using the MOE program.

Ligand	hAR interaction energy (kcal/mol)	Ligand–amino acid distance (Å)			
		N705	Q711	R752	T877
TBECH-α	−34.8	3.41	6.58	8.35	6.87
TBECH-β	−26.8	2.89	7.50	12.59	3.03
TBECH-γ	−35.3	4.10	4.60	6.04	3.26
TBECH-δ	−40.1	4.05	5.72	4.33	3.37
DHT	−53.8	2.07	1.87	3.93	1.58
Testosterone	−48.9	3.37	7.51	6.16	3.36

**Table 2:** Interaction energies and distances between the ligand and the key amino acids of the LNCaP AR (T877A) determined using the MOE program.

Ligand	LNCaP AR interaction energy (kcal/mol)	Ligand–amino acid distance (Å)			
		N705	Q711	R752	A877
TBECH-α	−37.6	4.43	4.00	5.27	5.41
TBECH-β	−41.2	5.20	3.89	6.62	5.47
TBECH-γ	−49.0	3.75	3.48	5.43	4.46
TBECH-δ	−43.0	3.75	3.37	4.22	4.27
DHT	−63.0	2.26	4.31	2.94	4.08
Testosterone	−82.5	2.84	3.32	2.73	4.73

**Table 3:** IC_50_ and EC_50_ values reported for binding/activation of steroid receptors by their natural ligands.

Receptor	IC_50_ (M)	EC_50_ (M)
ER	8.99 × 10^−10^^a^	1.76 × 10^−11^^b^
AR	7.7 × 10^−10^^c^	3.90 × 10^−10^^d^
PR	4.00 × 10^−9^^e^	2.30 × 10^−7^^f^
AhR	6.4 × 10^−11^^g^	4.88 × 10^−11^^h^

ER binds 17β-estradiol, AR binds DHT, progesterone receptor (PR) binds progesterone, and AhR binds TCDD.

a Data from Blair et al. (2000).

b Data from Pillon et al. (2005).

c Data from Cabeza et al. (2004).

d Data from Xu et al. (2005).

e Data from Viswanath et al. (2008).

f Data from Lenasi and Breskvar (2004).

g Data from Bonefeld-Jørgensen et al. (2007),

h Data from Westerink et al. (2008).

**Table 4:** RBA of ligands for human receptors.

	ER		AR		AhR	
Compound	RBA	RAF	RBA	RAF	RBA	RAF
Nonylphenol	0.037^a^	0.00016^b^	0.0523^c^	ND	ND	0.0003^d^
*o*,*p*′-DDT	0.0014^a^	0.0009^e^	0.0149^f^	0.0126^g^	ND	ND
Bisphenol A	0.0077^a^	0.0183^d^	0.0018^c^	ND	ND	ND
Vinclozolin	< 0.0009^a^	ND	0.0023^f^	0.0126^g^	ND	ND
Dieldrin	0.0019^h^	0.00001^b^	ND	ND	ND	ND
Aldrin	0.0029^h^	ND	0.007^f^	ND	ND	ND
TCDD	< 0.0002^g^	0.00012^g^	0.0013^g^	< 0.0013^g^	100	100
PCB 77	< 0.0003^a^	ND	ND	ND	ND	0.025^i^

Abbreviations: DDT, dichlorodiphenyltrichloroethane; ND, no relevant RBA/RAF for the ligand/receptor interaction has been reported to our knowledge; PCB 77, 3,3′,4,4′-tetrachlorobiphenyl; RAF, relative activation factor. ER binds 17β-estradiol, AR binds DHT, and AhR binds TCDD. All values are reported as percentage of binding/activation and were calculated according to Fang et al. (2003), with the values obtained with natural ligands of each receptor set to 100%.

a Data from Blair et al. (2000).

b Data from Pillon et al. (2005).

c Data from Scippo et al. (2004).

d Data from Bonefeld-Jørgensen et al. (2007).

e Data from Legler et al. (1999).

f Data from Fang et al. (2003).

g Data from Viswanath et al. (2008)

h Data from Sonneveld et al. (2005).

i Data from Zeiger et al. (2001).
